# Speed of performing complex movement tasks under decision-making conditions as a determinant of the tactical preparation level in kickboxers

**DOI:** 10.1038/s41598-024-53652-6

**Published:** 2024-02-06

**Authors:** Róża Gumienna, Weronika Machowska-Krupa, Jan Kosendiak

**Affiliations:** 1 Faculty of Physical Education and Sport, Wroclaw University of Health and Sport Sciences, 51-612 Wroclaw, Poland; 2 Department of Sports, Wroclaw University of Sports, 54-118 Wroclaw, Poland

**Keywords:** Neuroscience, Physiology

## Abstract

This study aimed to evaluate the relationship between the speed of performing a complex motor task carried out under conditions requiring decision-making and the sports level of the kickboxers being studied. The authors constructed a test involving a complex author’s kickboxing task that mirrored the competitive conditions during a sports fight. Forty-seven K-1 kickboxing Polish National Team members (37 men and 10 women) performed a specific series of strikes across three variants. The relationship between the speed of these complex movement tasks, performed under decision-making conditions, and the sports level of the kickboxers, was evaluated. Deciding to start or change an action in reaction to external stimuli significantly (*p* < 0.001) increased the total task completion time in the male and female subject groups. The time spent deciding to take action and the time spent on deciding on the action were not significantly different. Quick execution of complex tasks under decision-making conditions, such as selecting the appropriate technical and tactical action, can become a decisive factor in determining a sports result. Making decisions to start or change an action as a reaction to external stimuli significantly (*p* < 0.001) extended the total time of task execution in the studied group. However, the time spent deciding to start the action and the time devoted to deciding during the action did not differ significantly. Therefore, the tactical solution an athlete uses makes no difference, though they must perform it confidently and with minimal time loss.

## Introduction

Success in kickboxing depends on many factors, such as one’s shape, appropriately planned training processes involving anaerobic and aerobic changes, correctly chosen training means, and consequently, time devoted to regeneration, nutrition, supplementation, and working on the mental aspects of the sport^1^. Of course, technical movement skills and perfection when implementing fight tactics may prove crucial for the fight outcome. Tactical skills are defined as “the decisions and actions of players in the contest to gain an advantage over the opposing team or players”^2^^, p. 170^. Many researchers emphasize the role of tactics in sports activities, including Malkov and Kalashnikov^3^, Naglak^4^, and Czajkowski^5^. There are three options for tactical styles of fighting in martial arts: game, power, tempo^6^. Kozin et al.^7^ proposed psychophysiological and biomechanical research methods that reveal boxers' predispositions to a certain fighting style. “Psychophysiological features of boxers of different styles of fighting are reflected in the features of the technique of a direct blow. The lack of speed when tempo-style boxers are engaged in movement is supplemented and compensated by the high speed of movement. Play style boxers are characterized by high speed of movement at the very beginning of punching. Power style boxers are distinguished by the gradual development of movement speed”^8^^, p. 8^. Additionally, many scientists conduct statistical analysis on the use of a particular technique or time spent by athletes on offensive or defensive actions^9–12^ based on video recordings and a specially developed formula. Kickboxers are engaged in offensive actions more than defensive ones^13^. In order to use offensive or defensive techniques more effectively and improve specific sports skills in kickboxing, movement patterns must be understood^14–16^. Both types of action (offensive or defensive) must be consistent with the assumed goal^4^. In every case, the appropriate action should be chosen to maximize one’s capabilities and limit the opponent’s possibilities. The effectiveness of an athlete’s action depends primarily on the efficiency of their mental and motor functions. Mental functions include, among others, the ability for anticipation, evaluation, and decision-making, which affect the quality of tactical thinking^5^. Combat sports,are characterised by sudden environmental changes where each athlete must adapt to new situations in every moment^17^.

In kickboxing, the speed ability should be approached from a multifactorial perspective because it is not only about the speed of movement in the fighting position but also the speed of delivering a single punch or kick, the frequency of strikes, the speed of executing a series of strikes (combinations), the speed of a complex reaction to an emerging stimulus, the speed of the transition from defense to attack, and, of course, decision-making speed. In addition to actions and single techniques, a series of strikes are used that can last from several dozen milliseconds to several seconds^18^, which is related to the intermittent nature of fighting in kickboxing competitions at the international level. Moreover, the attack velocity for a forward karate punch was 2.7–3.0 mls and for a standing punch 1.9–2.1 mls^19^, and duration of the taekwondo athletes kick was approximately half a second^20^.Therefore, "during the training the fighters should pay special attention to constructing combinations of punches and kicks using hook high and roundhouse kick high techniques. Using proper techniques as well as numerous repetitions of the most effective techniques should be a part of any training of a kickboxing fighter”^21^^, p. 11^. Regardless of the decisions made by an athlete, they must be reached in the shortest possible time, at the right moment, and accurately in relation to the perceived stimulus and the tactical variant that they deem appropriate. It is worth noting that experienced competitors significantly shorten decision-making time and minimize reaction time by activating anticipatory factors^22^. During the fight, the athlete faces time and result pressure and opts for actions that can bring profit but are also burdened with the risk of failure. Therefore, it is necessary to stimulate the athlete’s decision-making in various conditions and situations during tactical preparation^23^.

The study analyzed the speed of performing a complex task by K-1 kickboxing athletes under decision-making conditions. It was assumed that an athlete representing a high sports level would spend less time making decisions during action, and therefore, the speed of performing a complex task under decision-making conditions would be higher. To verify this hypothesis, a complex motor task was constructed, consisting of an author’s kickboxing task that, to the highest possible degree, approximates the conditions of competition in a sports fight and, above all, takes into account aspects of speed, including reaction time, decisiveness over the choice of offensive and defensive tactical variants, and compliance in performing a motor task based on basic techniques, which should be habitual actions for high-class athletes.

## Materials and methods

### Participants

The study group consisted of representatives of the Polish senior national K-1 kickboxing team, including 37 men (weight category of 71.4 ± 10.4 kg; body height = 178.3 ± 8.8 cm; age 25.4 ± 4.2 years; training experience = 8.9 ± 4.4 years; total number of fights fought = 73.2 ± 43.7; percentage of fights won = 77.2 ± 10%) and ten women (weight category of 56.2 ± 6.4 kg; body height = 164.3 ± 4.7 cm; age = 26.3 ± 4.6 years; training experience = 8.1 ± 4.7 years; total number of fights fought = 79.1 ± 91.8; percent of fights won = 70.6 ± 11.5%). Thirty male athletes declared a tactical preference for offensive combat (81.1%) and seven for defensive (18.9%). Similar proportions were found in the women, with eight athletes declaring a tactical preference for offensive combat (80%) and two for defensive (20%).

### Methods

The study process involved an observation method that measured the time taken by the athletes to perform a complex motor task. The task was constructed to simulate typical actions performed by subjects during daily sports training and combat. The measurement tool was validated. In addition, all subjects signed a participant informed consent statement. The Senate Committee on Ethics of Scientific Research of the Wroclaw University of Health and Sport Sciences approved the study (34/2016). All experiments were performed in accordance with relevant named guidelines and regulations.

### Description of complex motor task performance under decision-making conditions

#### Organization of the study

A motor task test was constructed and conducted on the tatami mats in the afternoon (5–7 p.m.), with the temperature in the sports hall around 18–20 °C. The athletes entered the study as if they were starting a typical training session (adequately hydrated, two to three hours after a full meal, and wearing typical training outfits). Before the measurements, the subject performed an individual warm-up (15–20 min). As part of the warm-up, athletes used specialized training measures, such as shadow fighting and hitting a punching bag, allowing them to approach the task properly prepared and focused. During the measurements, the subject had to perform two series of A1 and A2 strikes on a punching bag (150 cm long, 45 cm in diameter, and weighing 55 kg) in three performance variations. The measurement was performed using the Fusion Sport SmartSpeed system (Technomex, Gliwice, Poland), which was synchronized with light signals that also delineated the test area. The light signals were 60 cm from the punching bag on its left and right sides for variant I. On the other hand, the light signals from variant II were located at the ends of the starting line (measuring 1 m left and right). The lights were 150 cm high and within sight of the participants.

#### Description of the study sample

**Figure 1:**
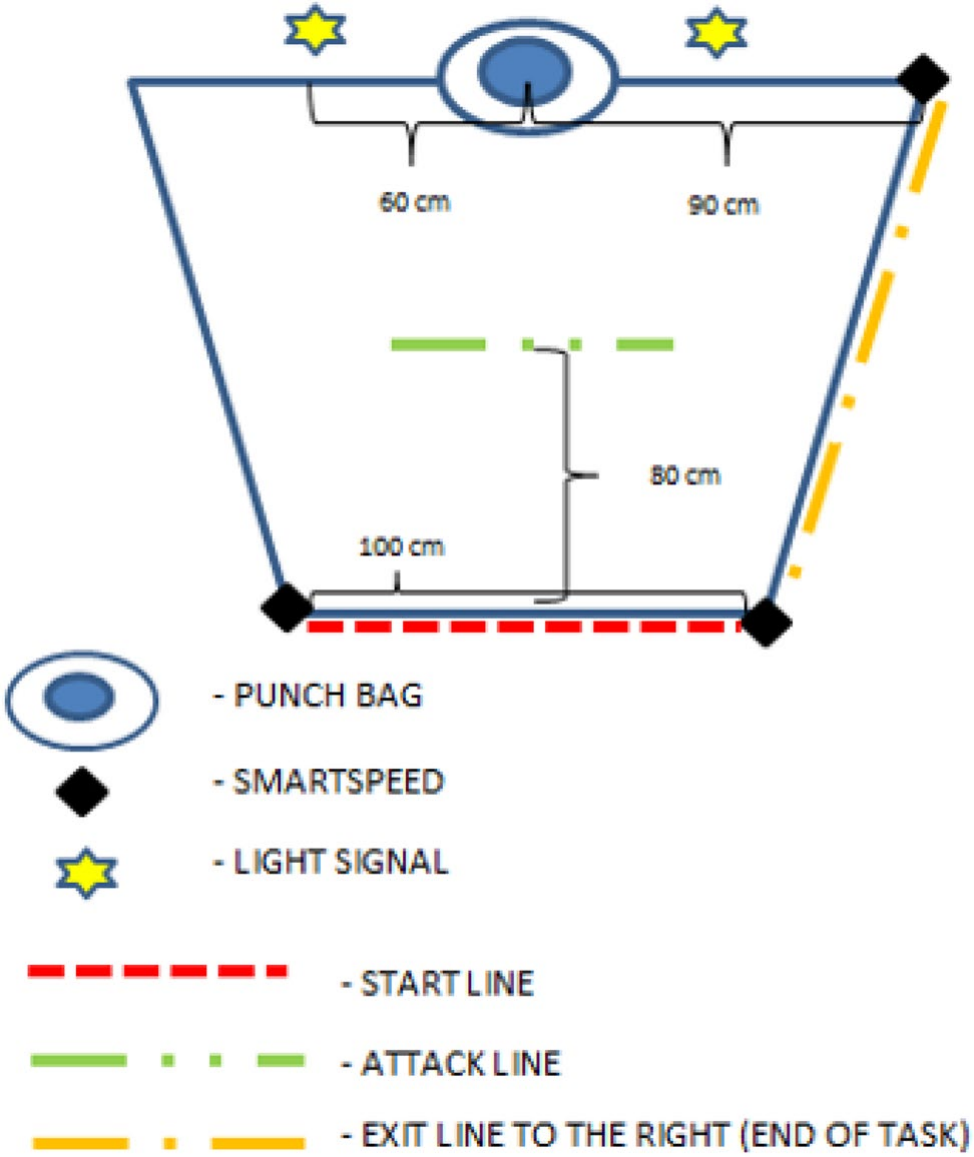
Test area schematic—Fusion Sport SmartSpeed system set-up (variant I).

The A1 and A2 strike series consisted of eight strikes performed using the arms (boxing punches) or legs (kicks). Both series A1 and A2 commenced with the same three strikes—a straight left punch (boxing punch), a straight right punch (boxing punch), and a right low-kick (kick). These series are differentiated by the remaining five strikes, which are different combinations of hand and foot techniques. For series A1, the five strokes consisted of a straight left punch, a straight right punch, a left sickle, a right low kick, and a right middle kick. The A2 series involved a straight left punch, right sickle, left hook, right low kick, and left middle kick. After performing the first three strikes, the subject would take a step back, crossing the line of attack, then continue the series by performing another five A1 or A2 strikes (simulating a second tempo fight). The two series were structured to represent the similar difficulty of their execution and utilized the right and left limbs (arms and legs). After the five strikes, the subject leaves the test area by crossing the exit line. A schematic of the test area and set-up of the Fusion Sport SmartSpeed device is shown in Fig. 1.

The participant performed the author’s kickboxing task in three variations. Variant I can be compared to the actions presented in defensive tactics, while variant II reflects the actions conducted by athletes who prefer to fight with offensive tactics. In contrast, decision-making was kept to a minimum in variant III.

Variant I (AD)—in this variant, the measurement involved the time to perform the A1 and A2 series, taking into account the time loss resulting from identifying the light signal (the light placed on the left pointed to the A1 series and the light on the right to the A2 series), which appeared after the first three strikes, and ordered the task completion (five consecutive strikes) according to the A1 and A2 schemes.

Variant II (AR)—in this variant, the measurement involved the time to perform the A1 and A2 series, taking into account the reaction time, i.e., the time to make a decision based on identifying the light signal and starting to perform the task. If the light on the left side came on, participants had to do the A1 series (three strokes back, forward to the attack line, then five A1 strokes), and if the light on the right side came on, they had to do the A2 series (three strokes back, forward to the attack line, and five A2 strokes).

Variant III (AB)—in this variant, the subject did not make any decision and performed the actions at a voluntary moment chosen by them. The object of measurement was only the execution time for series A1 and A2.

#### Athlete's personal data sheet

Each athlete completed an athlete personal data sheet before starting the test. The following information was obtained through the fighter personal data sheet questionnaire: fight position, major achievements, number of fights fought, number of fights won and lost (amateur and professional ones), body measurements, weight category and much more.

### Statistical analysis

The STATISTICA the version number 13.1 PL software package by StatSoft Poland (https://www.statsoft.pl/Zasoby/Do-pobrania/Wersja-probna-STATISTICA/) was used to process the data obtained during the measurement process. Statistical description was based on mean and standard deviation. For grouping (nominal) variables, simple counts and percentages were provided. Correlation between the variables were verified by r-Pearson coefficient. Duncan's tests were used as post-hoc tests. Homogeneity of variance and normality of distribution were verified. In doubtful cases, conclusions were verified by nonparametric tests, such as the Wilcoxon Test and The Mann–Whitney U-test.

## Results

**Figure 2:**
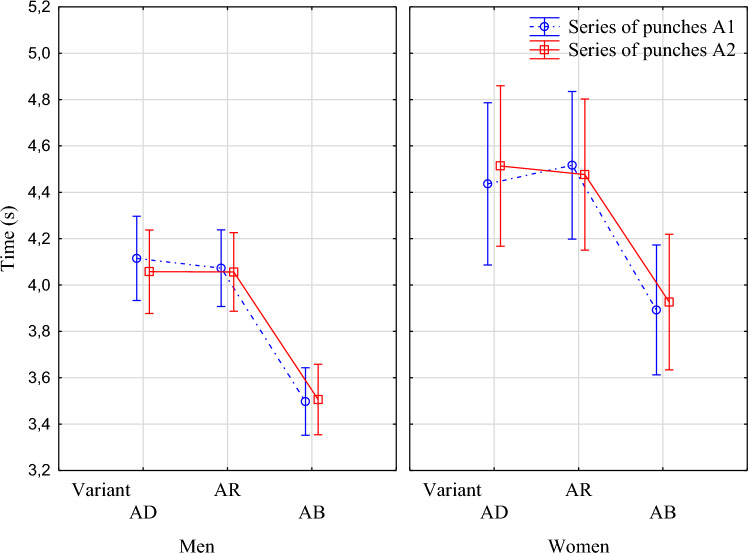
The time taken by men and women to perform a series of A1 and A2 strikes in each performance variation: I (AD), II (AR), III (AB).

The task completion times for all three variants were analyzed for series A1 and A2, with statistically significant differences in task performance shown between men and women in all variants for both series of strikes. Women spent more time performing the task, both when having to make decisions (AD and AR) and in situations with limited decision-making (the AB variants) (Fig. 2). The time to perform a series of A1 and A2 strikes by males and females in variants I and II, which include an additional decision-making moment, significantly increased the time taken to perform the task compared to variant III (p = 0.000) (Fig. 2).

It was also demonstrated that there was no significant time difference between males and females in the time to perform the A1 and A2 strike series in variant I and variant II (Tables 1 and 2).

**Table 1:** Duncan's post hoc test for performance on the complex movement of the A1 strike series for men and women in the variant I and II.

Gender	Variant	{1}	{2}	{3}	{4}
Men	I		0.578†	0.092†	0.046**
Men	II	0.578†		0.071†	0.033**
Women	I	0.092†	0.071†		0.295†
Women	II	0.046**	0.033**	0.295†	

**Significant at the 0.05 probability level; †Nonsignificant.

**Table 2:** Duncan's post hoc test for performance on the complex movement task of the A2 strike series for men and women in the variant I and II

Gender	Variant	{1}	{2}	{3}	{4}
Men	I		0.985†	0.025**	0.030**
Men	II	0.985†		0.029**	0.038**
Women	I	0.025**	0.029**		0.562†
Women	II	0.030**	0.038**	0.562†	

**Significant at the 0.05 probability level; †Nonsignificant.

**Figure 3:**
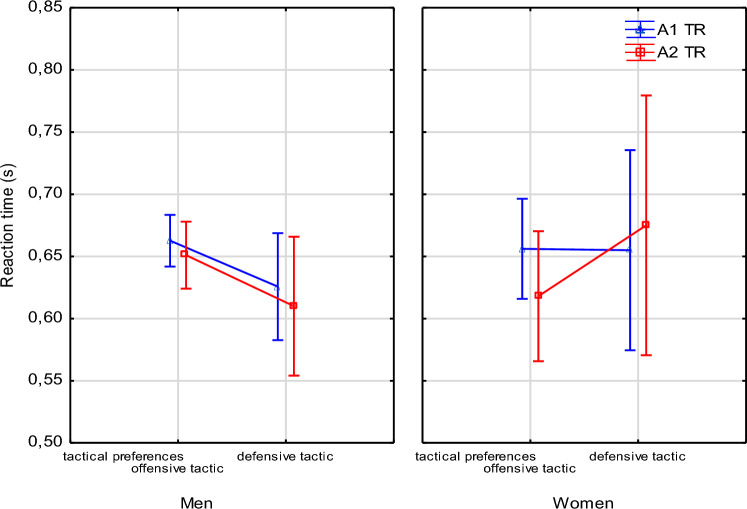
Difference in reaction time [TR] obtained during variant II measurement for A1. and A2 strike series and tactical preferences of male and female athletes.

In addition, there was no significant difference between the reaction time obtained during the execution of both series of strikes (A1, A2) in variant II (TR) in either male or female athletes who declared offensive or defensive fighting as the leading tactic in their sports career (Fig. 3).

When searching for factors determining sports mastery in kickboxing, we calculated the correlation of task performance time with weight category, body height, age, training experience, the total number of fights (amateur and professional), and the percentage of fights won for the group of men (Table 3).

**Table 3:** Correlations of variables obtained from athlete personal data sheet with time to complete a complex author’s kickboxing task for both series of strikes in three performance variants for the male group.

Series	Variant	Weight category (kg)	Body height (cm)	Age (years)	Training experience (years)	Total number of fights fought	Percentage of fights won (%)
A1	I	− 0.0040 p = 0.981†	0.0762 p = 0.654 †	− 0.1739 p = 0.303†	− 0.3065 p = 0.065†	− 0.4245 p = 0.009*	− 0.1729 p = 0.306†
	II	0.0492 p = 0.772†	0.0515 p = 0.762†	− 0.1527 p = 0.367†	− 0.2337 p = 0.164†	− 0.2913 p = 0.080†	− 0.2522 p = 0.132†
	III	0.2762 p = 0.098†	0.2151 p = 0.201†	0.1650 p = 0.329†	0.0498 p = 0.770†	− 0.0308 p = 0.857†	− 0.1004 p = 0.554†
A2	I	0.0057 p = 0.973†	0.0517 p = 0.761†	− 0.1448 p = 0.392†	− 0.2634 p = 0.115†	− 0.3851 p = 0.019*	− 0.2653 p = 0.112†
	II	− 0.0029 p = 0.986†	0.0056 p = 0.974 †	− 0.1640 p = 0.332†	− 0.2267 p = 0.177†	− 0.3291 p = 0.047*	− 0.2044 p = 0.225†
	III	0.1017 p = 0.549†	0.1297 p = 0.444†	− 0.0772 p = 0.650†	− 0.1714 p = 0.310†	− 0.1048 p = 0.537†	− 0.1380 p = 0.415†

*Significant at the 0.000 and lower probability level; †Nonsignificant.

The correlation coefficient values obtained (Table 3) indicate that the performance time of some variant I in the A1 series and variant I and II in series A2 correlated with the total number of fights fought, i.e., with the experience of these fighters. The female group showed a significant correlation between the percentage of fights won and task completion time in variant II in the A1 series and variant I in the A2 series. In addition, the timing of the A1 and A2 in variants I and II significantly correlated with the total number of fights fought and training experience (Table 4).

**Table 4:** Correlations of variables obtained from athlete’s personal data sheet with time to complete a complex author’s kickboxing task, for both series of strikes in three performance variants for the female group.

Series	Variant	Weight category (kg)	Body height (cm)	Age (years)	Training experience (years)	Total number of fights fought	Percentage of fights won (%)
A1	I	− 0.0141 p = 0.969†	0.0812 p = 0.824†	0.0805 p = 0.825†	− 0.7356 p = 0.015*	− 0.6744 p = 0.032*	− 0.5976 p = 0.068†
	II	0.0988 p = 0.786†	0.1637 p = 0.651†	0.0588 p = 0.872†	− 0.7577 p = 0.011*	− 0.6988 p = 0.025*	− 0.6534 p = 0.040*
	III	− 0.4711 p = 0.169†	− 0.3998 p = 0.252†	0.6432 p = 0.045*	− 0.2505 p = 0.485†	− 0.3096 p = 0.384†	− 0.4217 p = 0.225†
A2	I	0.1488 p = 0.682†	0.2259 p = 0.530†	− 0.0241 p = 0.947†	− 0.7463 p = 0.013*	− 0.6361 p = 0.048*	− 0.6577 p = 0.039*
	II	0.0230 p = 0.950†	0.1438 p = 0.692†	0.1075 p = 0.768†	− 0.7374 p = 0.015*	− 0.6601 p = 0.038*	− 0.5970 p = 0.068†
	III	− 0.2143 p = 0.552†	− 0.1698 p = 0.639†	0.4804 p = 0.160†	− 0.4582 p = 0.183†	− 0.4556 p = 0.186†	− 0.5487 p = 0.100†

*Significant at the 0.000 and lower probability level; †Nonsignificant.

In the male group, reaction time correlated significantly with the percentage of fights won, though there were no significant correlations between reaction time and the other variables (Table 5). In the female group, there were no clear correlations between series A1 and A2 in reaction time (variant II) or for the other variables.

**Table 5:** Correlations of A1 and A2 series in reaction time with variables: weight category, body height, age, training experience, total number of fights, percentage of fights won for men's group.

Gender	Series	Variant	Weight category (kg)	Body height (cm)	Age (years)	Training experience (years)	Total number of fights fought	Percentage of fights won (%)
Men	A1	II	− 0.1832 p = 0.278†	− 0.0697 p = 0.682†	− 0.0094 p = 0.956†	− 0.2340 p = 0.163†	− 0.1757 p = 0.298†	− 0.4638 p = 0.004*
Women			− 0.0099 p = 0.978†	− 0.3495 p = 0,322†	− 0.0235 p = 0.949†	0.2890 p = 0.418†	− 0.0318 p = 0.930†	− 0.0062 p = 0.986†
Men	A2	II	− 0.1670 p = 0.323†	− 0.1080 p = 0.525†	− 0.0953 p = 0.575†	− 0.1541 p = 0.363†	− 0.2516 p = 0.133†	− 0.4729 p = 0.003*
Women			0.7305 p = 0.016*	0.6847 p = 0.029*	− 0.5437 p = 0.104†	− 0.6283 p = 0.052†	− 0.3769 p = 0.283†	− 0.3169 p = 0.372†

*Significant at the 0.000 and lower probability level; †Nonsignificant.

## Discussion

Based on scientific research, it is very difficult to find specialized tests to verify the level of fitness-related and tactical preparation of kickboxers. In most cases, the scale of difficulty of performing a mental-physical task during official combat sports competitions is much greater in comparison with laboratory psychophysical tests^12^. Therefore authors used their own measurement tool, which was validated. Having considered the foregoing, for the purposes of this study, a complex author’s kickboxing task was included the performance times for a series of strikes (A1, A2) in three variants of execution, reaction time from the variant II, the number of mistakes made by the athletes during the task, the results obtained from the athletes' personal data sheet. Statistical calculations of the time to complete the author’s kickboxing task in three variants showed a difference between men and women in terms of the presented motor skill level, and more specifically: the manifestation of speed. The longer time needed by women to perform a motor task maybe the results of sexual dimorphism. It manifests itself not only in the morphological and physiological properties of the organism, but also in physical performance, leading to variations in physical fitness^24^. On average, when compared to men, women are characterized by lower strength, speed, endurance and agility, as well as higher flexibility, precision and accuracy of movement^25^. In addition, Iwańska^26^ showed that the level of speed and power in women also depends on the menstrual cycle. It affects changes in speed levels in 66% and power levels in 7.5% of the total women studied, with individual women responding to menstrual cycle presence in typical ways. Ferreira & Vencesbrito^27^ noted in karate athletes an association with a difference in the pattern of neuromuscular coordination controlling movement during strikes between men and women. It was despite the fact that a similar kinematic pattern was expected due to the same discipline practiced by both groups.

In our study, a difference in author’s kickboxing task completion time was observed between the three variants. If there was a stimulus forcing a change or correction of action over its course, the time of task performance increased significantly. The situation was similar when the athlete had to make a decision to initiate an action as a reaction to an external stimulus. "Dondersa (1868) was the first to find that complex processes take about 0.1 s longer than simple reactions. Similarly, a study by Klapp and Erwin (1976) showed that additional information delays reaction time by 100–150 ms" [after: ^18^^, p. 18^]. This relationship was not demonstrated in this study because the athletes spent statistically the same amount of time making decisions and performing the task from variant I, where there was a complex reaction (choice between two possibilities), and the task from variant II with a simple reaction, when the athlete had to make a decision as quickly as possible, and react to a single stimulus, and the only correct response was to start the task by crossing the starting line. This relationship is also reflected in the papers by Ryguła and Borysiuk^28^, as well as Sadowski^29^ and Żukowski^30^. Occasionally, outstandingly talented athletes (in karate, taekwondo and fencing) have manifested complex reaction times that are strongly similar to simple reaction times, i.e., responses with a significant degree of automaticity.

Tomczak^31^ pointed out the relationship between reaction time with selection, and performance effectiveness in combat sports, as manifested through the style of diverse actions. Defensive athletes make more decisions between alternative courses of actions, demonstrating shorter reaction times with selection. Athletes with an offensive style make quicker decisions on an action-reaction basis, without alternatives (they attack more often). It is worth noting that athletes fighting using offensive tactics outnumber those who fight with defensive tactics. In our study, defensive fighting was preferred by 18.92% of men and 20% of women. Krupalija et al.^32^ and Ouergui et al.^13^ emphasize the importance of offensive combat for winning in kickboxing. Kickboxing fighters winning their fights used more offensive actions, compared to defensive ones. It is accepted that defensive combat is more difficult, requires different decision making, tremendous focus and composure, and only a few outstanding athletes achieve mastery by using it.

Numerous scientific paper authors^33–40^ report that performance speed, regardless of the sport, is affected by athletic level, which can be determined by many factors, for example: experience, training experience (over time), motivation level, past athletic performance, or physical conditions. The author of this paper showed that task completion time correlates with the athletic level of kickboxers. Athletes presenting higher sports level complete the task faster. In men, the sports level is reflected in the place occupied by the number of fights (experience). In case of women, the sports level is also reflected in higher number of fights and longer training experience. Numerous studies^5,30,41–44^ report that athletes' experience significantly affects performance in sport. The difference between advanced and less advanced athletes is not necessarily related to the value of simple reaction times, but to more complex factors, such as complex reaction time, anticipatory ability, attentional focus, and rapid analysis of situations. Experienced athletes are presumed to significantly reduce decision-making time by reducing reaction time and consequently the time to complete the entire task by activating anticipatory factors^22^. Anticipation affects the reduction of latent reaction time and motor phase, in sports with open movement habits^45^. The use of technical skills, mastered to at least good level, significantly improves and reduces the action time, which in turn can provide an advantage in a situation where they need to be used in a sport fight or self-defense settings^46^. In some sports, we are able to pinpoint the preferred physical conditions of athletes that can facilitate their pursuit of athletic mastery. Nevertheless, in combat sports, it is possible to compensate for certain disadvantages connected with the parameters of the body structure, and to use the strongest sides of the athlete through appropriately selected combat tactics. Such a phenomenon was observed, e.g., by Lech et al.^47^ in judo, where athletes' technical and tactical activity varies according to their physique. Competitors with lower body height, in comparison to the weight category in which they compete, present greater activity in attack and a greater variety of techniques. Medium-height athletes make more hand throws compared to tall athletes, who are more likely to choose leg throws. In contrast, when observing taekwondo athletes during a tournament, Miller^48^ clearly found that the physique of the winners and losers did not differ significantly, it was the condition preparation that played a key role in the athletic outcome.

The test of a complex author’s kickboxing task presented in the study was verified in terms of the task performance speed as a determinant of the tactical preparation of athletes. The authors hope that the obtained study results will provide an additional source of knowledge for coaches and athletes striving for sports mastery.

## Conclusions

The time to perform a motor task in both the men’s and women’s groups was significantly lengthened (*p* < 0.001) when a stimulus appeared during an action, forcing a change or correction. Deciding to start an action as a response to external stimuli significantly (*p* < 0.001) extended the total time to perform the task in both the men’s and women’s groups. Furthermore, the time spent deciding to start an action and the time spent making decisions during the action were not significantly different between males and females.

In men, the time to perform some variants of the author’s kickboxing task significantly correlated (*p* < 0.05) with athletic level, expressed as the total number of fought fights. Meanwhile, no significant correlation was shown between the prolongation of the time to perform the motor task resulting from decision-making and training experience, the percentage of fights won, weight category, or height. In women, the time to perform a motor task resulting from decision-making significantly correlated (*p* < 0.05) with athletic level, expressed as the total number of fights fought and training experience. In variants I and II, in which an element of decision-making appeared, female athletes with more training experience and a higher number of fights performed more difficult tasks faster. No significant correlation was shown between prolonging the time to perform the motor task resulting from decision-making and the percentage of fights won, weight category, or height.

Thus, whether a kickboxer uses defensive or offensive tactics during a fight bear no significance, whereas their perfect, consistent action, with as little time wasted as possible, is important. Therefore, technical and tactical action schemes should be refined in the training process. However, during the fight, many stimuli flow to the athlete, and they must quickly perceive, select, and make a decision regarding their tactical response. Additionally, the athlete predicts the opponent’s actions by anticipating their movements through insightful observation. As such, coaches should create variable conditions and enable independent decision-making for athletes in the training process. The simplest method, of course, is sparring, or training techniques that only impose the initial sequence of movements, where the athlete responds to offensive actions of their training partner in any way they choose in the further part of the training session. However, it is worth equipping oneself with additional tools, including auxiliary means, non-contact (without contact with a partner, which can be performed independently), and forcing the athlete to shorten their reaction time, an example of which is the research tool proposed by the authors.

## Data Availability

The datasets used during the current study are available from the corresponding author on reasonable request.
